# Image-based assessment of the clinical effects of medical training on scapulothoracic muscle group for shoulder joint mechanical stability

**DOI:** 10.1097/MD.0000000000044286

**Published:** 2025-09-12

**Authors:** Boming Liu, Baixun Wang, Kang Tian, Guannan Chen, Weiguo Zhang

**Affiliations:** a Department of Sports Medicine, First Hospital of Dalian Medical University, Dalian, China; b Department of Arthrology and Sports Medicine, Jilin Municipal People’s Hospital, Jilin, Jilin, China; c Medical Imaging Cent of Jilin Municipal People’s Hospital, Jilin, Jilin, China.

**Keywords:** humeral head, joint stability, medical training therapy, scapulothoracic muscles, shoulder

## Abstract

**Background:**

This study assessed the therapeutic effectiveness of medical training therapy (MTT) in reducing the upward migration of the humeral head during shoulder abduction, focusing on enhancing the scapulothoracic muscles to improve shoulder joint stability. The primary outcome measure was the vertical displacement of the humeral head’s center of rotation before and after treatment.

**Methods:**

From January 15, 2023 to January 3, 2024, 63 patients with rotator cuff tears were randomly assigned to either the experimental (n = 32) or control (n = 31) group. Additionally, 49 healthy volunteers were included as a healthy control group. All participants underwent X-ray imaging of shoulder abduction at 0° and 30° before and after the treatment. The experimental group received a 3-month regimen of conservative management combined with MTT targeting the scapulothoracic muscles, whereas the control group received only conservative treatment. An image-based measurement technique quantified the relative upward migration of the humeral head.

**Results:**

Before treatment, the relative upward migration was similar between the experimental (7.06%) and control groups (7.30%, *P *= .57). After treatment, the experimental group exhibited a significant reduction in upward migration (4.93%) compared with that in the control group (7.02%, *P *< .001). By contrast, the control group displayed only a minimal decrease from 7.30% to 7.02%.

**Conclusion:**

MTT significantly reduces the upward migration of the humeral head during shoulder abduction, leading to improved rehabilitation outcomes, whereas conventional conservative treatment has minimal effect on migration reduction. By enhancing shoulder joint stability and potentially slowing the progression of rotator cuff degeneration, MTT represents a valuable adjunct to conservative management strategies.

## 1. Introduction

Rotator cuff tears represent a common shoulder injury in the general population.^[1]^ The incidence of rotator cuff injuries increases with age,^[2]^ rising from 5% to 10% in individuals aged 20 years and younger to 30% to 35% among those aged 60 to 70 years and peaking at 60% to 65% in individuals aged 80 years and older.^[3]^ Nonsurgical treatments are often prioritized for managing established rotator cuff tears when appropriate,^[4,5]^ whereas surgical interventions are typically reserved for acute or severe cases.^[6]^ During shoulder joint movement,^[7]^ dynamic stabilization driven by the rotator cuff and surrounding muscles ensures that the humeral head and glenoid center remain aligned.^[8]^

Injury to the supraspinatus weakens its stabilizing function,^[9]^ allowing the deltoid to shift the humeral head upward.^[10]^ This misalignment can result in increased friction against the acromion,^[11]^ worsening tendon damage, and potentially initiating a cycle of injury that can lead to tendon rupture.^[12]^ Studies indicated that during shoulder abduction in healthy individuals, the rotational center of the humeral head remains stable relative to the glenoid. However, in patients with rotator cuff tears, the early phase of abduction disrupts the force couple balance between the supraspinatus and deltoid muscles,^[13]^ impairing the supraspinatus’s ability to stabilize the humeral head.^[14]^ This instability causes the rotational center of the humeral head to shift upward.^[15]^ By measuring and quantifying this rotational instability, clinicians can better evaluate shoulder joint function and assess treatment outcomes for patients.

Studies revealed that scapular training is effective in restoring joint stability and improving treatment outcomes in patients recovering from rotator cuff injuries.^[16]^ Early scapular training promotes shoulder functional recovery and improves rehabilitation outcomes in patients with rotator cuff injuries.^[17]^ Grip strength training has also been demonstrated to alleviate shoulder pain in patients with primary subacromial impingement.^[18]^ Additionally, systematic rehabilitation exercises have proven beneficial for reducing symptoms of subacromial impingement.^[19]^ For patients with rotator cuff tears, medical training therapy (MTT) targeting the scapulothoracic muscles in the shoulder complex can help reduce upward movement of the humeral head during abduction and restore force couple balance.^[20,21]^

However, limited research exists on the potential of MTT to stabilize rotator cuff injuries by targeting scapulothoracic depressor muscles to delay or treat rotator cuff tears. Furthermore, few imaging-based studies have confirmed the link between rotator cuff tears and instability in the center of rotation during shoulder movement.^[22,23]^ This study aimed to quantitatively assess the therapeutic effectiveness of MTT in reducing the upward migration of the humeral head during shoulder abduction. The therapy focused on enhancing the scapulothoracic subtension muscle group to improve shoulder joint stability. Unlike previous approaches, our protocol integrates a comprehensive rehabilitation system that enhances clinical feasibility alongside an objective assessment of treatment efficacy through standardized imaging metrics. This combination provides a more practical, measurable, and clinically relevant framework for evaluating patient outcomes.

## 2. Methods

### 2.1. Selection of patients

From January 15, 2023 to January 3, 2024, 63 patients with rotator cuff tears were randomly selected from the outpatient clinic of the Department of Joint and Sports Medicine at our hospital to participate in this randomized controlled trial. The study protocol was approved by the Ethics Committee of Dalian Medical University. Informed consent was obtained from the participants for the publication of this study.

Sample size was determined based on a power analysis. Using G*Power software, we set the parameters as follows: a medium effect size (Cohen d = 0.5) referenced from relevant literature, an alpha level of 0.05 (2-tailed), and a statistical power of 80%. The analysis indicated a minimum requirement of 58 patients. Considering potential dropouts (estimated at 10%), a total of 63 patients were enrolled to ensure sufficient power for the primary outcome analysis.

The inclusion criteria were as follows:

1. Age between 18 and 65 years.
2. Symptoms persisting for more than 1 month.
3. No limitation in active or passive range of motion of the shoulder joint during physical examination, with no significant adhesions observed.
4. Positive results for both the job test and hug resistance test during physical examination.
5. Diagnosis of a full-thickness supraspinatus tear confirmed by magnetic resonance imaging. A full-thickness rotator cuff tear was identified on at least 2 consecutive MRI slices with a slice interval of 3 mm.

The exclusion criteria were as follows:

1. History of shoulder fracture in the affected limb.
2. History of shoulder surgery in the affected limb.
3. Presence of brachial plexus injury or other neurological disorders.

The 63 patients were randomly assigned to either the experimental (32 patients) or the control group (31 patients). Additionally, 49 healthy volunteers were selected as an independent control group to support the evaluation of the therapeutic effects of the rehabilitation exercise developed in this study. The baseline characteristics of patients and healthy individuals are shown in Table 1.

**Table 1:** Baseline characteristics of patients and healthy individuals.

Age					
	Number	Mean age	*t* score	*P*	
Healthy	49	55.65 ± 8.34	0.229	.796	
Control	31	54.42 ± 10.03			
Experimental	32	54.56 ± 9.08			

Sex					
	Number	Female	Male	χ^2^	*P*
Healthy	49	29	20	0.168	.920
Control	31	19	12		
Experimental	32	18	14		

Participants were randomly assigned to the treatment groups using a computer-generated random sequence prepared by a statistician independent of the research team. Group allocation was concealed in sequentially numbered, sealed, and opaque envelopes. After obtaining informed consent from the participants, a research assistant not involved in patient recruitment opened the envelopes and performed the allocation to ensure allocation concealment. To minimize bias, participants, intervention providers, and outcome assessors were all blinded to group assignments.

Prior to the experiment, all participants underwent X-ray to assess the upward movement of the humeral head during shoulder abduction.

During the experimental phase, subjects in the experimental group received a 3-month comprehensive treatment regimen, which included conservative management along with targeted MTT focused on the scapulothoracic depressor muscle group of the shoulder complex. Subjects in the control group received only conservative treatment, which consisted of rest for the affected limb, oral nonsteroidal anti-inflammatory drugs, physical therapy, and intra-articular glucocorticoid injections.^[24]^

The conservative management protocol included pharmacological therapy, physical therapy modalities, and intra-articular injections.

#### 2.1.1. Pharmacological management

Patients received oral nonsteroidal anti-inflammatory drug therapy, specifically ibuprofen 200 mg administered twice daily with meals.

#### 2.1.2. Physical therapy modalities

Infrared thermotherapy was applied locally to the affected shoulder joint. Each session lasted 15 minutes and was administered twice daily. A safety distance of 30 cm between the infrared lamp and the skin surface was maintained throughout the treatment.

#### 2.1.3. Intra-articular injection regimen

Patients received ultrasound-guided intra-articular glenohumeral injections under strict aseptic conditions. The injection solution consisted of triamcinolone acetonide 40 mg (depot formulation), 5 mL of 1% lidocaine (equivalent to 86.5 mg), and 5 mL of normal saline as a diluent. Injections were administered once weekly for 2 consecutive weeks. Following each injection, patients were instructed to immobilize the shoulder for 24 hours and were provided with guidance on activity modification.

After 3 months, both groups underwent follow-up X-ray to assess the recovery of joint stability and motor function.

### 2.2. Image-based evaluation method

An anteroposterior X-ray of the shoulder joint was performed with the shoulder joint was positioned laterally, ensuring that the anterior and posterior edges of the glenoid were aligned and centered as much as possible for optimal visualization. To ensure the reliability of radiographic assessments, standardized imaging protocols were implemented. Patients were positioned in an upright standing posture, with the center of the shoulder joint placed 10 cm from the detector backplate and a source-to-patient distance of 50 cm. All imaging procedures were performed by the same certified radiology technician to maintain consistency. This optimized protocol enables sensitive detection of humeral head rotational center migration during joint movement, permitting quantitative evaluation of glenohumeral instability.

**Figure 1.**
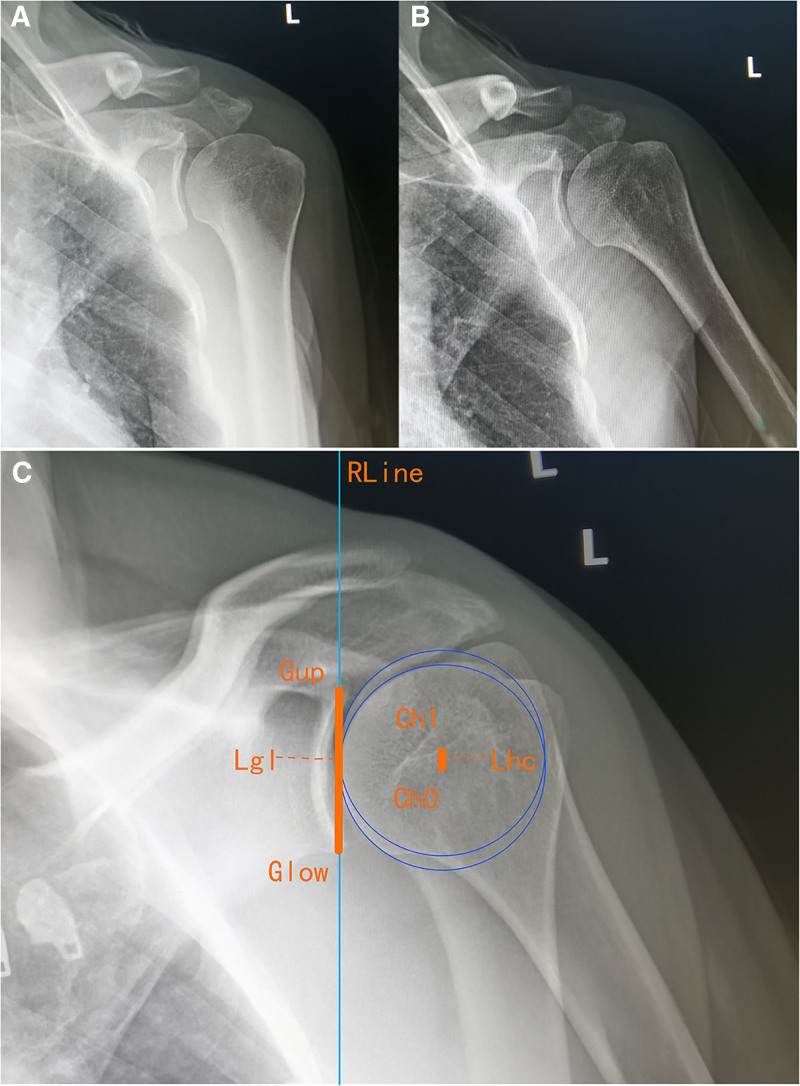
An image-based measurement technique for the evaluation of the upward movement of the humeral head. Anteroposterior (AP) X-rays of the shoulder joint in abduction positions of 0° (A) and 30° (B). (C) The calculation of the relative magnitude of the humeral head’s upward movement (Rlhg = Lhc/Lgl).

In healthy individuals, the rotational center of the humeral head relative to the glenoid remains stable during shoulder abduction, particularly from 0° to 30°.^[25,26]^ By contrast, in patients with rotator cuff tears, disruption of force couple balance during early abduction (0–30°) weakens the supraspinatus muscle’s stabilizing effect, causing joint instability and an upward shift in the humeral head’s rotational center.^[27]^ Therefore, the shoulder abduction positions at 0° and 30° were imaged (Fig. 1A and B).

An image-based measurement technique was developed to quantitatively evaluate the upward movement of the humeral head as follows:

1. The images at 0° and 30° abduction were aligned by moving and rotating the 30° abduction image. Adjustments to the size of only 1 image were prohibited to ensure alignment of the scapular glenoid between the 2 images (Fig. 1C). A new image was created from the overlapping images.
2. A vertical reference line (RLine) was drawn along the lateral edge of the glenoid fossa (anatomical landmark: the outer cortical margin of the glenoid cavity) (Fig. 1C).
3. The RLine placement criteria were strictly defined as follows: it must be parallel to the long axis of the glenoid and tangent to its lateralmost point. The new image was rotated so that the superior and inferior edges of the glenoid fossa (labeled as anatomical landmarks Gup and Glow, respectively) were aligned to touch RLine. The distance between Gup (superior glenoid rim) and Glow (inferior glenoid rim) (Lgl) represented the height of the scapula.
4. A circle was drawn tangent to the humeral head articular surface in the 0° abduction position, with its center labeled as Ch0. A duplicate circle was created and moved to the 30° abduction position, tangent to the humeral head articular surface, with the center labeled as Ch1. The distance between Ch0 and Ch1 (Lhc) represented the upward movement of the humeral head.
5. The length ratio between Lhc and Lgl, denoted as Rlhg = Lhc/Lgl, was calculated. This ratio reflects the relative magnitude of the humeral head’s upward movement independent of the humeral head size, which can vary among patients.

### 2.3. MTT method

The patients in the experimental group underwent MTT targeting the scapulothoracic depressor muscles of the shoulder complex for 3 months. The training was initiated in the subacute stage following the acute phase. The therapy focused on strengthening the muscles of the rotator cuff, scapula, and scapulothoracic region. Improving the strength and neuromuscular coordination of the rotator cuff muscles can enhance muscle endurance.^[28,29]^ The training load was individualized according to each patient’s baseline muscle strength, with the initial intensity set at 50% of the maximum voluntary capacity for each patient. Progression was assessed using both subjective and objective criteria, including patient-reported ease of movement execution, periodic isokinetic strength assessments to quantify muscular improvement, and monitoring of pain reduction during functional shoulder activities. This multimodal, evidence-based approach ensured a gradual increase in training intensity while prioritizing patient tolerance and promoting optimal functional recovery. The training protocol included multiple steps.

**Figure 2.**
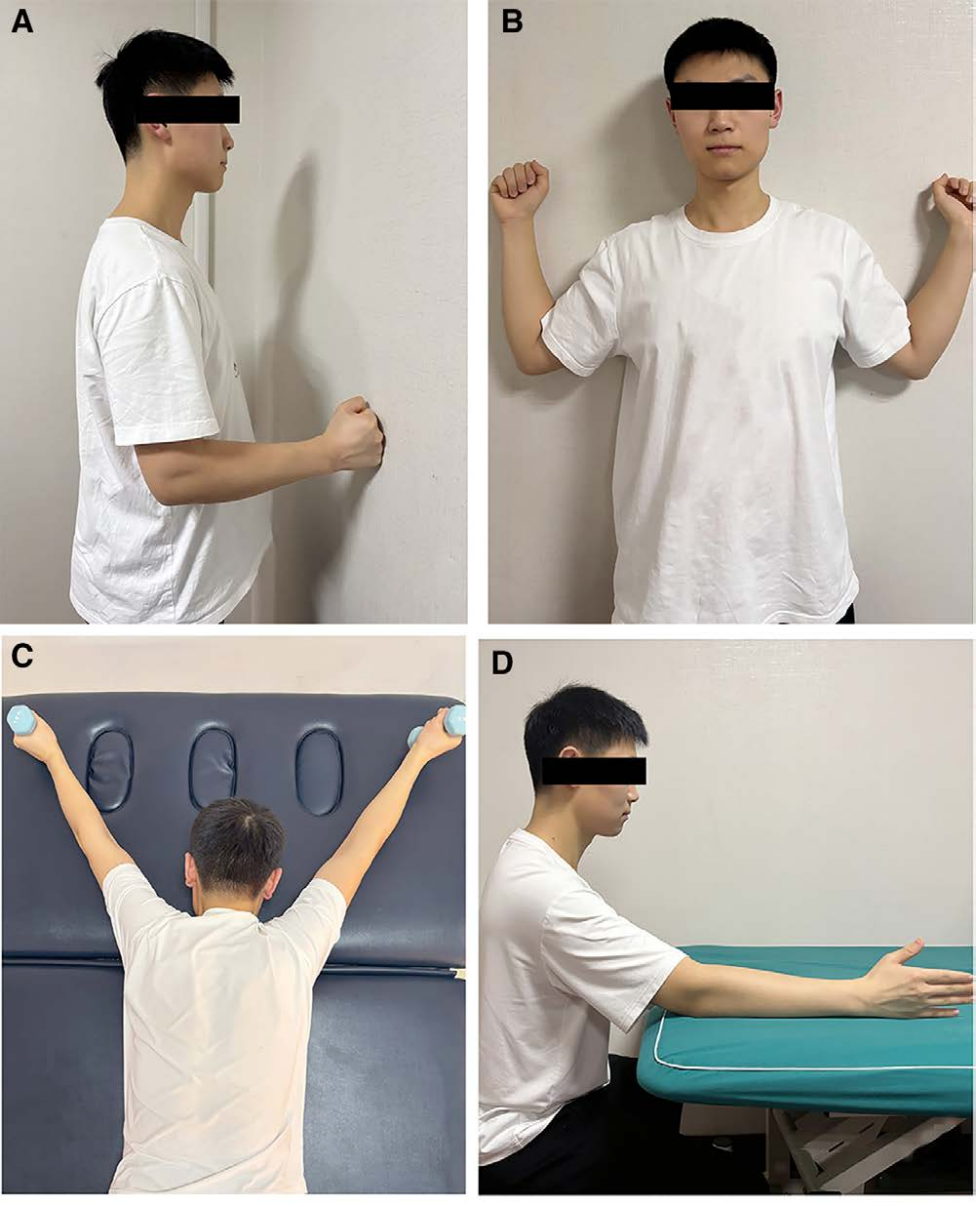
Joint stretching training exercises. (A) Isometric muscle contraction, (B) wall W-shape posture, (C) prone Y-shape posture, and (D) seated serratus anterior exercise.

1. Joint stretching training: in a standing position and holding 1 to 2-kg dumbbells, the patient gently pulled the joint capsule through heavy traction while keeping the shoulder relaxed. The stretch was held for 15 to 30 seconds, with 10 repetitions per set and 1 to 2 sessions per day.
2. Isometric muscle contraction (Fig. 2A): in a standing position with the upper limbs extended and the body bent forward 15°, the hands pressed against a wall, maintaining continuous force for 15 to 30 seconds. The muscle length remained unchanged, but tension increased during contraction. This exercise was also performed with the upper limbs extended backward by 15°.
3. Wall W-shape posture exercise (Fig. 2B): participants stood with their bodies against the wall, their upper arms abducted, and their elbows bent to form a “W” shape. The palms faced outward, and the shoulders, elbows, and hands were kept close to the wall. The arms were slowly slide up and down for 5 to 10 seconds per repetition, with 10 repetitions per set and 3 to 4 sets per day.
4. Prone Y-shape posture exercise (Fig. 2C): Participants laid prone with their arms extended in a “Y” shape, forming fists with their thumbs up. The participants exhaled and contracted their back muscles to lift their chests off the ground, after which they inhaled and lowered their backs to the ground. Each set included 10 repetitions, and 3 to 4 sets were performed per day.
5. Seated serratus anterior exercise (Fig. 2D): participants sat upright with their shoulders bent to 90°, keeping the back straight. The scapula was used to drive the upper arm forward and backward, focusing on scapular movement. Participants exhaled for 3 to 5 seconds, after which they inhaled and returned slowly to the initial position. Each set included 10 repetitions, and 3 to 4 sets were performed per day.

This training protocol aimed to improve shoulder joint stability and function by targeting key muscle groups involved in shoulder movement and stability.

After the subacute stage, if no significant pain is present in the shoulder joint, training to maintain and improve the range of motion, as well as enhance muscle strength, can be initiated. Active movement training is encouraged to further increase the joint’s range of motion. Progressive resistance training targeting the rotator cuff and scapular muscles has been reported to restore normal scapulohumeral rhythm, supporting dynamic stabilization of the scapula, and glenohumeral joint.^[21,30]^ This training reduces the risk of subacromial impingement and helps restore maximal muscle strength.^[31]^ The training protocol included several exercises.

**Figure 3.**
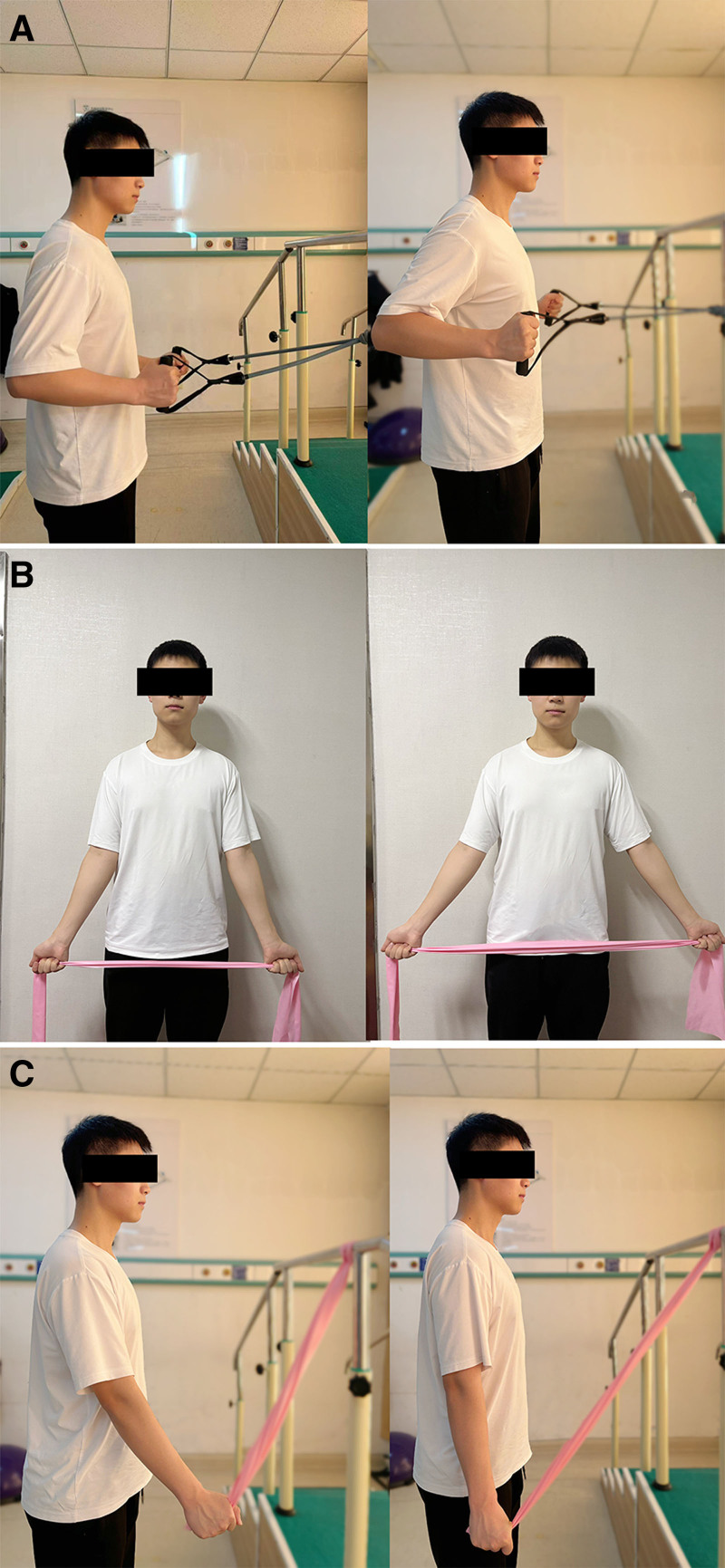
Resistance training exercises. (A) Resistance rowing exercise, (B) resistance low chest expansion, and (C) resistive straight arm pulldown.

1. Resistance rowing exercise (Fig. 3A): participants stood with the elastic band fixed in front, holding it with both hands and elbows bent at 90°. The back was kept straight and tight. Participants exhaled as they extended their arms backward, inhaled for 3 to 5 seconds, and then slowly returned to the starting position. Each set included 10 repetitions, and 3 to 4 sets were performed per day.
2. Resistance low chest expansion exercise (Fig. 3B): participants stood and grasped the elastic band with both palms facing upward. The shoulders were abducted to 45°, and participants exhaled while extending their arms backward against resistance, inhaled for 3 to 5 seconds, and then slowly returned to the starting position. Each set included 10 repetitions, and 3 to 4 sets were performed per day.
3. Resistive straight arm pulldown (Fig. 3C): participants stood with the elastic band fixed overhead and grasped it with their arms extended upward. Participants exhaled as they pulled the band downward, resisting the movement, inhaled for 3 to 5 seconds, and then slowly returned to the starting position. Each set included 10 repetitions, and 3 to 4 sets were performed per day.

This protocol aimed to enhance scapulohumeral rhythm and joint stability while building strength in key muscle groups essential for shoulder function and rehabilitation.

Strengthening external rotator exercises helps reduce pressure in the subacromial space.^[32]^ Agrebi utilized ballistic training with a specialized throwing apparatus and demonstrated significant improvements in shoulder rotational kinetics among throwers, offering valuable evidence-based protocols and reference data for implementing our investigation.^[33]^ Improving the initial strength of the rotator cuff muscles, extending motion beyond the mechanical impingement angle, and enhancing scapular muscle strength are crucial for maintaining scapular stability and normal scapular movement.^[34]^ Additionally, these exercises stimulate muscle fiber growth,^[35]^ promoting muscle hypertrophy. The climbing-specific TEST protocol demonstrated excellent intertrial and intersession reliability (intraclass correlation coefficient = 0.96–1.00) in quantifying lateral reach asymmetry. However, the absolute symmetry index exhibited limited sensitivity (coefficient of variation = 41.98%), indicating that bilateral difference metrics should be interpreted with caution.^[36]^ These findings align with the biomechanical model proposed by Dhahbi, suggesting that muscle-specific MTT improves joint stability by minimizing compensatory movements.^[37]^ Based on this evidence, we recommend incorporating VR-based perceptual motor training to further improve cognitive–biomechanical adaptation.

**Figure 4.**
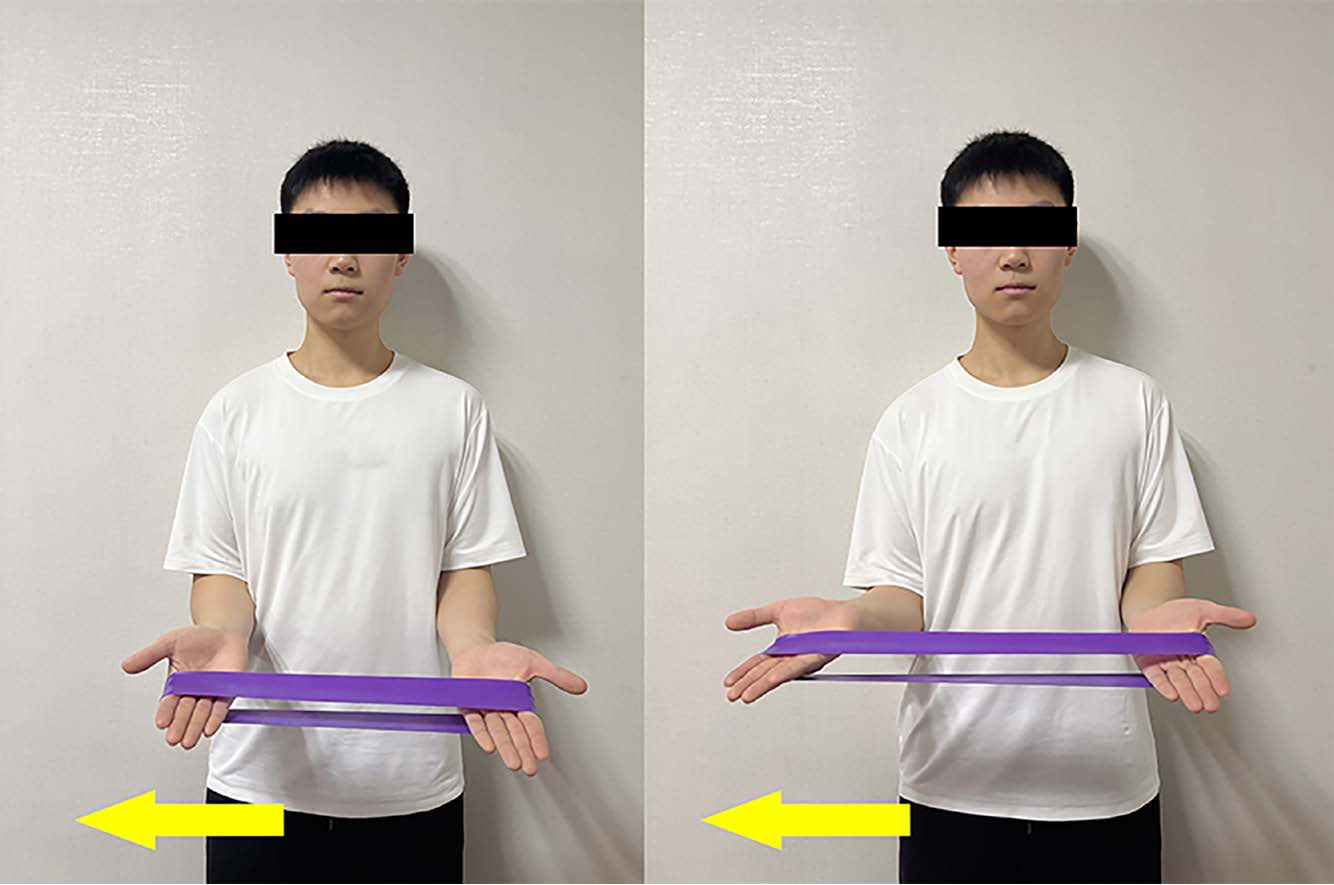
Lateral external rotation with resistance.

The training protocol for these exercises focused on lateral external rotation with resistance (Fig. 4). Patients stood with their elbows bent at 90° while holding an elastic band. They kept their arms close to their body with their palms facing upward. Patients exhaled as they rotated their arms outward against resistance and inhaled while holding the stretch for 3 to 5 seconds before slowly returning to the starting position. Each patient performed 10 repetitions per set, completing 3 to 4 sets daily or until muscle fatigue. Consistent with established reliability standards for power assessment,^[38]^ our protocol incorporated multiple test trials to ensure measurement stability. Furthermore, based on the findings of Turki, who demonstrated that dynamic stretching effectively prepares athletes for competition-specific demands, we recommend prioritizing dynamic stretching during rehabilitation warm-ups to optimize functional readiness.^[39]^

After 3 months of training, muscle balance during shoulder abduction exercises was restored. Post-training X-rays were taken to assess the degree of upward humeral head movement, providing quantitative data on the improvement of joint stability.

### 2.4. Statistical analysis

The imaging measurements of the shoulder joint were evaluated, and the resulting data were statistically analyzed using SPSS 18.0 software (SPSS Inc., Chicago). An independent-samples *t* test was conducted to compare the data between the rotator cuff tear and healthy individual groups. Additionally, an independent-samples *t* test was used to compare the results between the experimental and control patient groups before and after treatment. A paired-samples *t* test was performed to analyze the differences within each patient group before and after treatment. In this study, to adhere to the intention-to-treat principle (including all randomly assigned participants), missing data (including those from dropouts) were handled by carrying forward the last recorded observation. This retains all randomized participants in the analysis, minimizing bias from dropout and enhancing validity, as detailed in the CONSORT diagram.

## 3. Results

In total, 63 patients with rotator cuff tears were selected for the study, and 49 healthy individuals were evaluated through imaging. The relative magnitude of the humeral head’s upward movement, reflected by Rlhg, was 7.17% (95% CI = 6.67–7.57) in patients, compared with 2.3% (95% CI = 2.17–2.41) in healthy individuals (*P* < .05, Table 2).

**Table 2:** Comparison of results between the experimental and control groups before and after treatment.

Group	Number	Mean Rlhg (before treatment, %)	95% CI	t score	*P*	Mean Rlhg (after treatment, %)	95% CI	t score	*P*
Control	31	7.30 ± 1.72	6.63–8.00	0.57	.57	7.02 ± 1.53	6.37, 7.55	5.21	<.001
Experimental	32	7.06 ± 1.55	6.36–7.56			4.93 ± 1.60	4.44, 5.62	4.96	<.001
Healthy individuals	49	2.30 ± 0.47	2.17–2.41	−19.97	<.001				
Patients	63	7.17 ± 1.64	6.67–7.57						

Data are presented as percentages.

CI = confidence interval.

An independent-samples *t* test was used to assess the differences between the 2 patient groups before and after treatment. Prior to treatment, Rlhg was similar between the experimental (7.06%, 95% CI = 6.36–7.56) and control groups (7.30%, 95% CI = 6.63–8.00, *P* > .05). However, after treatment, the experimental group demonstrated a significantly lower value (4.93%, 95% CI = 4.44–5.62) than the control group (7.02%, 95% CI = 6.37–7.55), as presented in Table 2 (*P* < .05).

**Figure 5.**
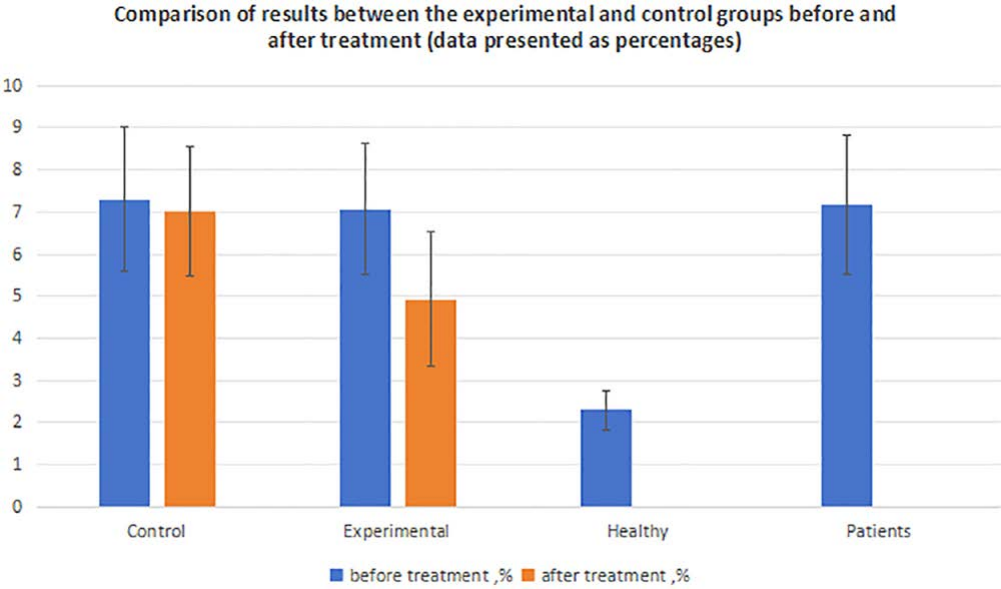
Evaluation of shoulder joint stability before and after treatment in the experimental group, control group, and healthy individuals.

A paired-samples *t* test was performed to evaluate within-group changes before and after treatment. In the experimental group, medical training reduced Rlhg from 7.06% to 4.93% (*P* < .05). By contrast, conventional management in the control group led to a minimal reduction from 7.30% to 7.02% (Table 2). Neither medical training nor conventional therapy fully restored the healthy reference value of 2.30% measured in the healthy control group (*P* < .05, Fig. 5). Safety monitoring revealed no serious adverse events during the study. Approximately 5% of participants experienced mild, self-limiting symptoms, such as fatigue, which resolved spontaneously without intervention.

## 4. Discussion

This study primarily focused on patients with complete supraspinatus tendon tears. Imaging measurements revealed significant upward migration of the humeral head. Mean Rlhg in the patient group was 7.17%, which was 3-fold higher than the mean value of 2.30% observed in the healthy control group (Table 2). The supraspinatus muscle plays a key role in initiating shoulder abduction and works in conjunction with the deltoid muscle to form a force couple. This force couple helps maintain the rotation of the humeral head in place during abduction. Effective rehabilitation protocols are essential for restoring balance within the glenohumeral–scapular kinetic chain to mitigate superior humeral head translation and prevent rotator cuff impingement.^[40]^ The dynamic stability of the glenohumeral joint relies on the coordinated activation of rotator cuff muscles in synergy with the surrounding shoulder musculature.^[41]^

In patients with rotator cuff tears, the rupture of the supraspinatus muscle weakens its ability to compress the humeral head, and consequently, the deltoid muscle’s pull on the humeral head leads to upward migration of the center of rotation during shoulder abduction.

In this study, MTT was used to target the scapular muscles within the shoulder complex. Because of the action of the rotator cuff, the humeral head is consistently compressed onto the glenoid during shoulder abduction, counteracting the force exerted by the deltoid muscle that pulls the humeral head upward. This mechanism helps prevent the humeral head from being pulled upward and impinging on the acromion. Imaging data obtained 3 months post-treatment revealed a significant reduction in Rlhg during shoulder abduction from 7.06% before treatment to 4.93% after treatment, representing a 30% decrease. By contrast, the traditional treatment approach only reduced the value from 7.30% to 7.02%, reflecting a modest 4% decrease. The imaging data from both treatment groups demonstrate that MTT is significantly more effective than traditional treatments in reducing Rlhg during shoulder abduction. It is important to note that the duration of post-treatment observation is crucial. In this study, a 3-month observation period was used, whereas some published studies typically observed patients for 3 to 7 weeks after treatment,^[42]^ which often leads to the conclusion that the therapeutic effect is inadequate. This is likely because shoulder function and pain symptoms do not improve sufficiently in such a short period, indicating that a longer duration is needed to fully assess rehabilitation outcomes.

This study had several limitations. First, although both the MTT and traditional rehabilitation methods achieved some improvement in reducing humeral head displacement in patients with rotator cuff tears, neither approach fully restored the displacement to levels observed in healthy individuals. Additionally, the long-term therapeutic outcomes of MTT were not fully explored because of the limited follow-up period. The study also did not investigate the potential influence of sex and age on treatment outcomes, which was a constraint imposed by the sample characteristics. Furthermore, an in-depth experimental analysis of the biomechanics underlying MTT was not conducted, which could provide valuable insights for optimizing this treatment approach. The study was subject to potential selection bias, as patients with higher compliance and motivation to adhere to the study protocol might have been preferentially enrolled. Such participants were likely to exhibit proactive healthcare-seeking behaviors and positive attitudes, which could have contributed to more favorable treatment outcomes. To mitigate this, we implemented 2 specific strategies during recruitment: we used standardized inclusion/exclusion criteria applied uniformly by trained research assistants to avoid subjective enrollment decisions; we explicitly included a diverse range of participants across different age groups and disease severities, as documented in the “Participants” section, to reduce over-representation of highly motivated individuals. Despite these efforts, residual selection bias cannot be fully excluded, and this should be considered when interpreting the findings. Additionally, the use of incomplete blinding might have introduced measurement bias. The relatively short follow-up period (limited to 3 months) precluded evaluations of long-term treatment effects beyond this timeframe, which limits the generalizability of the findings regarding the durability of outcomes. Future studies with extended follow-up (e.g., 6–12 months) are warranted to better assess the long-term efficacy and sustainability of the intervention. Furthermore, the absence of patient-reported outcomes restricted insight into subjective treatment experiences. Future research should aim to address these limitations through more rigorous study designs.

## 5. Conclusion

The MTT method developed in this study significantly reduces the upward migration of the humeral head during shoulder abduction, leading to improved rehabilitation outcomes. By contrast, conventional conservative treatment had only a minimal effect on reducing this value. MTT exercises, which target the scapulothoracic depressor muscles, help prevent the humeral head from being pulled upward, contributing to more favorable clinical outcomes during shoulder abduction. This noninvasive, easily implementable training approach offers a practical conservative alternative, potentially reducing the need for surgical intervention while supporting postoperative recovery. Future research should aim to establish detailed biomechanical models of the shoulder to better evaluate the contributions of individual muscle groups during movement, enabling the development of personalized rehabilitation protocols.

## Author contributions

**Data curation:** Boming Liu.

**Formal analysis:** Boming Liu.

**Funding acquisition:** Boming Liu.

**Investigation:** Baixun Wang.

**Methodology:** Baixun Wang.

**Resources:** Guannan Chen.

**Software:** Guannan Chen.

**Validation:** Weiguo Zhang.

**Visualization:** Weiguo Zhang.

**Writing – original draft:** Kang Tian.

**Writing – review & editing:** Kang Tian.

## References

[1] Bedi A Bishop J Keener J. Rotator cuff tears. Nat Rev Dis Primers. 2024;10:8. 38332156 10.1038/s41572-024-00492-3

[2] Newton JB Fryhofer GW Rodriguez AB Kuntz AF Soslowsky LJ. Mechanical properties of the different rotator cuff tendons in the rat are similarly and adversely affected by age. J Biomech. 2021;117:110249. 33486263 10.1016/j.jbiomech.2021.110249 PMC7920936

[3] Varacallo MA El Bitar Y Sina RE Mair SD. Rotator cuff syndrome. 2024 Mar 5. In: StatPearls. Treasure Island (FL): StatPearls Publishing; 2025. 30285401

[4] Zhang C Wang J Wang L. The effect of physiotherapy in rotator cuff injury patients with platelet-rich plasma: study protocol of a non-randomized controlled trial. BMC Musculoskelet Disord. 2021;22:292. 33743650 10.1186/s12891-021-04171-2 PMC7981950

[5] Shepet KH Liechti DJ Kuhn JE. Nonoperative treatment of chronic, massive irreparable rotator cuff tears: a systematic review with synthesis of a standardized rehabilitation protocol. J Shoulder Elbow Surg. 2021;30:1431–44. 33276163 10.1016/j.jse.2020.11.002

[6] Patel M Amini MH. Management of acute rotator cuff tears. Orthop Clin North Am. 2022;53:69–76. 34799024 10.1016/j.ocl.2021.08.003

[7] Dong W Goost H Lin XB. Treatments for shoulder impingement syndrome: a PRISMA systematic review and network meta-analysis. Medicine (Baltim). 2015;94:e510. 10.1097/MD.0000000000000510 PMC4602475 25761173

[8] Aranha L Eapen C Patel VD Prabhakar AJ Hariharan K. Muscle fatigue response of rotator cuff muscles in different postures. Arch Orthop Trauma Surg. 2023;143:3191–9. 36305967 10.1007/s00402-022-04650-8 PMC10191943

[9] Sakaki Y Taniguchi K Katayose M Kura H Okamura K. Effects of shoulder abduction on the stiffness of supraspinatus muscle regions in rotator cuff tear. Clin Anat. 2022;35:94–102. 34668243 10.1002/ca.23800 PMC9298298

[10] Graichen H Stammberger T Bonél H. Three-dimensional analysis of shoulder girdle and supraspinatus motion patterns in patients with impingement syndrome. J Orthop Res. 2001;19:1192–8. 11781023 10.1016/S0736-0266(01)00035-3

[11] Reddy AS Mohr KJ Pink MM Jobe FW. Electromyographic analysis of the deltoid and rotator cuff muscles in persons with subacromial impingement. J Shoulder Elbow Surg. 2000;9:519–23. 11155306 10.1067/mse.2000.109410

[12] Croen BJ Carballo CB Wada S. Chronic subacromial impingement leads to supraspinatus muscle functional and morphological changes: evaluation in a murine model. J Orthop Res. 2021;39:2243–51. 33336819 10.1002/jor.24964

[13] Knighton TW Chalmers PN Sulkar HJ Aliaj K Tashjian RZ Henninger HB. Anatomic total shoulder glenoid component inclination affects glenohumeral kinetics during abduction: a cadaveric study. J Shoulder Elbow Surg. 2022;31:2023–33. 35550434 10.1016/j.jse.2022.03.028 PMC9481675

[14] Çalik M Utlu DK Demirtaş A Canbora MK Erdil ME Düzgün I. Is shoulder joint position sense affected in partial and full-thickness supraspinatus tears? Int Orthop. 2023;47:1021–9. 36719444 10.1007/s00264-023-05702-3

[15] Steenbrink F de Groot JH Veeger HE van der Helm FC Rozing PM. Glenohumeral stability in simulated rotator cuff tears. J Biomech. 2009;42:1740–5. 19450803 10.1016/j.jbiomech.2009.04.011

[16] Zhang M Zhou J Zhang Y Zhang X Chen J Chen W. Influence of scapula training exercises on shoulder joint function after surgery for rotator cuff injury. Med Sci Monit. 2020;26:e925758. 33116073 10.12659/MSM.925758 PMC7607672

[17] Yu M Tang Z. The effect of early scapular training on shoulder joint function after surgery for rotator cuff injuries: a retrospective study. Ann Ital Chir. 2024;95:1231–9. 39723510 10.62713/aic.3632

[18] AlAnazi A Alghadir AH Gabr SA. Handgrip strength exercises modulate shoulder pain, function, and strength of rotator cuff muscles of patients with primary subacromial impingement syndrome. Biomed Res Int. 2022;2022:9151831. 36082154 10.1155/2022/9151831 PMC9448609

[19] Abdulla SY Southerst D Côté P. Is exercise effective for the management of subacromial impingement syndrome and other soft tissue injuries of the shoulder? A systematic review by the Ontario Protocol for Traffic Injury Management (OPTIMa) Collaboration. Man Ther. 2015;20:646–56. 25920340 10.1016/j.math.2015.03.013

[20] Roy JS Moffet H Hébert LJ Lirette R. Effect of motor control and strengthening exercises on shoulder function in persons with impingement syndrome: a single-subject study design. Man Ther. 2009;14:180–8. 18358760 10.1016/j.math.2008.01.010

[21] Worsley P Warner M Mottram S. Motor control retraining exercises for shoulder impingement: effects on function, muscle activation, and biomechanics in young adults. J Shoulder Elbow Surg. 2013;22:e11–9. 10.1016/j.jse.2012.06.010 PMC3654498 22947240

[22] Savoie A Mercier C Desmeules F Frémont P Roy JS. Effects of a movement training oriented rehabilitation program on symptoms, functional limitations and acromiohumeral distance in individuals with subacromial pain syndrome. Man Ther. 2015;20:703–8. 25907145 10.1016/j.math.2015.04.004

[23] Azzoni R Cabitza P Parrini M. Sonographic evaluation of subacromial space. Ultrasonics. 2004;42:683–7. 15047367 10.1016/j.ultras.2003.11.015

[24] Hopewell S Keene DJ Marian IR. Progressive exercise compared with best practice advice, with or without corticosteroid injection, for the treatment of patients with rotator cuff disorders (GRASP): a multicentre, pragmatic, 2 × 2 factorial,randomised controlled trial. Lancet. 2021;398:416–28. 34265255 10.1016/S0140-6736(21)00846-1 PMC8343092

[25] Endo K Ikata T Katoh S Takeda Y. Radiographic assessment of scapular rotational tilt in chronic shoulder impingement syndrome. J Orthop Sci. 2001;6:3–10. 11289583 10.1007/s007760170017

[26] Akhtar A Richards J Monga P. The biomechanics of the rotator cuff in health and disease - a narrative review. J Clin Orthop Trauma. 2021;18:150–6. 34012769 10.1016/j.jcot.2021.04.019 PMC8111677

[27] Ishikawa H Smith KM Wheelwright JC. Rotator cuff muscle imbalance associates with shoulder instability direction. J Shoulder Elbow Surg. 2023;32:33–40. 35961497 10.1016/j.jse.2022.06.022

[28] Beaudreuil J Lasbleiz S Aout M. Effect of dynamic humeral centring (DHC) treatment on painful active elevation of the arm in subacromial impingement syndrome. Secondary analysis of data from an RCT. Br J Sports Med. 2015;49:343–6. 23525552 10.1136/bjsports-2012-091996

[29] Galetta MD Keller RE Sabbag OD. Rehabilitation variability after rotator cuff repair. J Shoulder Elbow Surg. 2021;30:e322–33. 33418088 10.1016/j.jse.2020.11.016

[30] Beaudreuil J Lasbleiz S Richette P. Assessment of dynamic humeral centering in shoulder pain with impingement syndrome: a randomised clinical trial. Ann Rheum Dis. 2011;70:1613–8. 21623001 10.1136/ard.2010.147694

[31] Thigpen CA Shaffer MA Gaunt BW Leggin BG Williams GR Wilcox RB 3rd. The American Society of Shoulder and Elbow Therapists’ consensus statement on rehabilitation following arthroscopic rotator cuff repair. J Shoulder Elbow Surg. 2016;25:521–35. 26995456 10.1016/j.jse.2015.12.018

[32] Gallagher BP Bishop ME Tjoumakaris FP Freedman KB. Early versus delayed rehabilitation following arthroscopic rotator cuff repair: a systematic review. Phys Sportsmed. 2015;43:178–87. 25797067 10.1080/00913847.2015.1025683

[33] Agrebi B Dhahbi W Abidi H. Isokinetic peak torque improvement and shoulder muscle ratios imbalance correction after specific strength training on a new ballistic throwing device: a randomized controlled trial. J Sport Rehabil. 2024;33:423–36. 39032923 10.1123/jsr.2023-0253

[34] Nikolaidou O Migkou S Karampalis C. Rehabilitation after rotator cuff repair. Open Orthop J. 2017;11:154–62. 28400883 10.2174/1874325001711010154 PMC5366376

[35] Sciarretta FV Moya D List K. Current trends in rehabilitation of rotator cuff injuries. SICOT J. 2023;9:14. 37222530 10.1051/sicotj/2023011 PMC10208043

[36] Čular D Dhahbi W Kolak I. Reliability, sensitivity, and minimal detectable change of a new specific climbing test for assessing asymmetry in reach technique. J Strength Cond Res. 2021;35:527–34. 29939903 10.1519/JSC.0000000000002694

[37] Dhahbi W Chamari K Chèze L Behm DG Chaouachi A. External responsiveness and intrasession reliability of the rope-climbing test. J Strength Cond Res. 2016;30:2952–8. 26849786 10.1519/JSC.0000000000001367

[38] Dhahbi W Chaouachi A Cochrane J Chèze L Chamari K. Methodological issues associated with the use of force plates when assessing push-ups power. J Strength Cond Res. 2017;31:e74–e74.

[39] Turki O Dhahbi W Padulo J. Warm-up with dynamic stretching: positive effects on match-measured change of direction performance in young elite volleyball players. Int J Sports Physiol Perform. 2019;15:528–33. 31693996 10.1123/ijspp.2019-0117

[40] Goetti P Denard PJ Collin P Ibrahim M Hoffmeyer P Lädermann A. Shoulder biomechanics in normal and selected pathological conditions. EFORT Open Rev. 2020;5:508–18. 32953136 10.1302/2058-5241.5.200006 PMC7484714

[41] Johnson DJ Tadi P. Multidirectional shoulder instability. 2023 Jul 3. In: StatPearls. Treasure Island (FL): StatPearls Publishing; 2025. 32491658

[42] Boudreau N Gaudreault N Roy JS Bédard S Balg F. The addition of glenohumeral adductor coactivation to a rotator cuff exercise program for rotator cuff tendinopathy: a single-blind randomized controlled trial. J Orthop Sports Phys Ther. 2019;49:126–35. 30501388 10.2519/jospt.2019.8240

